# Obliterative Hepatocavopathy: The “Acrobatic Dolphin” Mimic as Endovascular Procedure Guide

**DOI:** 10.31729/jnma.v64i293.9278

**Published:** 2026-01-31

**Authors:** Ashwin Garg, Krantikumar Rathod

**Affiliations:** 1 Department of Radiology, Hamdard Institute of Medical Sciences and Research, Tughlaqbad, New Delhi, India; 2 Department of Radiology, Seth G S Medical College and KEM Hospital, Parel, Mumbai, India

**Keywords:** *hepatic congestion*, *hepatocavopathy*, *inferior venacava obstruction*, *sign*

## Abstract

Obliterative hepatocavopathy, also known as membranous obstruction of the inferior venacava, is a frequently underdiagnosed vascular condition, seen particularly in low socioeconomic populations in India, Nepal and other parts of Asia. This viewpoint presents a distinctive radiographic analogy- the “Acrobatic Dolphin” mimic-observed during venacavography in a patient with membranous inferior venacava obstruction. The dolphin-like configuration, best appreciated in the left anterior oblique view, featured a conical beak-shaped membrane formed by flow dynamics that served both as a diagnostic clue and as an endovascular procedural guide for successful membranotomy. Moreover, recognition of such radiological signs provides powerful mnemonics that enhance clinical education.

## INTRODUCTION

Obliterative hepatocavopathy,^1^ often termed fibrous Membranous inferior venacava (IVC) obstruction is a frequent yet underdiagnosed condition, particularly prevalent among low socio-economic population in Nepal, India and other Asian countries. It typically presents with symptoms and signs related to chronic hepatic congestion. Despite advances in cross-sectional imaging, catheter venography remains the gold standard for both diagnosis and therapeutic planning, as it delineates the morphology of the obstructing membrane and guides endovascular intervention.^2^

Beyond its diagnostic role, venography often reveals unique visual analogies that enhance recognition and understanding of complex vascular morphology. Here, we describe a distinctive venographic appearance resembling an “acrobatic dolphin,” observed in a patient with membranous IVC obstruction. This visual “mimic” serves both as an educational aid and as a practical landmark during endovascular membranotomy.

## THE “ACROBATIC DOLPHIN” MIMIC

**Figure 1:**
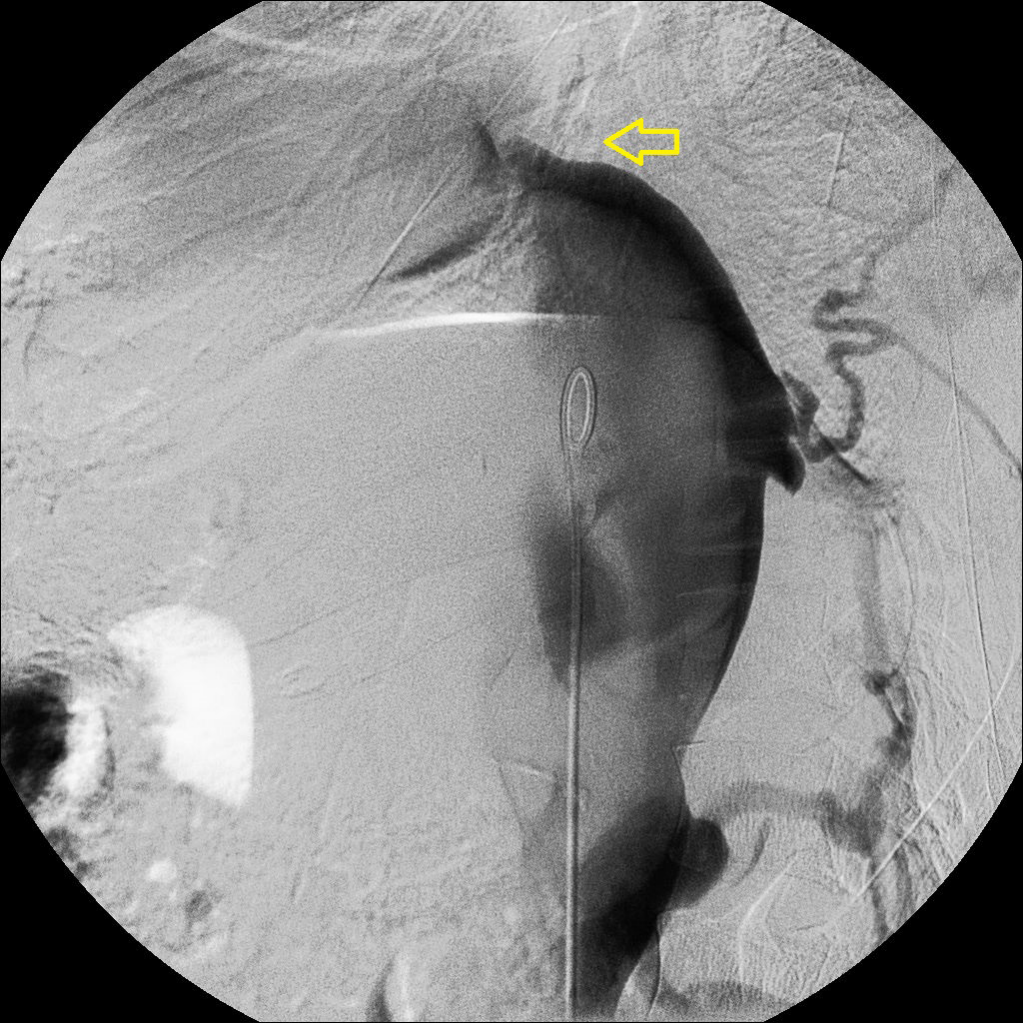
Inferior venacavogram in the left oblique view showing contrast outlining the dilated intrahepatic IVC, forming the body of a dolphin. Collateral veins mimic fins, and the conical obstruction (arrow) resembles the beak.

Catheter venography performed for evaluation of chronic hepatic congestion in suspected Budd-Chiari syndrome demonstrated complete obstruction in the retrohepatic segment of the IVC with proximal dilatation and multiple paravertebral collaterals. In the left anterior oblique projection, with patient in supine position, the dependent layering of radiocontrast formed a silhouette that strikingly resembled an acrobatic dolphin on cine fluoroscopy:

- The dilated IVC represented the trunk of the dolphin.
- The origin of branching paravertebral collateral veins, to bypass the blockage, appeared as fins.
- The conical membranous obstruction resembled a short beaklike snout (Figure 1).
- The undilated infrahepatic IVC resembled the slender part of dolphin.

This visual analogy, while informal, proved clinically useful. The pointed conical “beak” served as a landmark for planning subsequent endovascular membranotomy.

**Figure 2:**
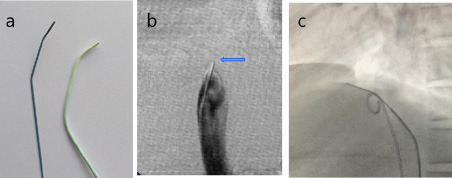
Endovascular Membranotomy: (a) The stiff end of a 260 cm Amplatz super stiff guide wire (Boston Scientific) is shaped to match the distal curves of a Headhunter angiography catheter. (b) Substracted image: The wire (arrow) is positioned within the “dolphin snout” and the membranous obstruction is punctured, directed medially in the frontal view. (c) Non substracted image: In the lateral view, puncture is directed anteriorly. Note the pigtail angiography catheter in the IVC, introduced via the contralateral femoral vein for intermittent check venography.

**Endovascular membranotomy:** The stiff end of a 0.035” guide wire was shaped with two angulations matching the curve of a 5F Headhunter angiography catheter (Figure 2). The wire, coaxially placed within the catheter, was advanced to the level of obstruction and parked within the narrowed conical “beak” zone. Under frontal and lateral fluoroscopic guidance, controlled puncture of the membrane was performed from the concave hepatic side, with the wire tip directed antero-medially to align with the IVC axis. Small intermittent injections of contrast were used to confirm correct orientation until the right atrium was entered. The patient was regularly monitored for chest discomfort, serving as a safety check of unintended wire deviation. A characteristic, sudden “give-way” sensation was felt once the membrane was punctured.

**Figure 3:**
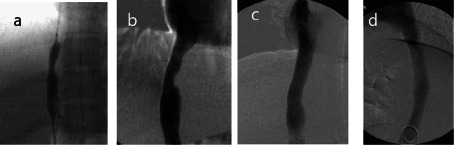
Post membranotomy: (a) Pre dilatation was performed by 8 × 40mm balloon, (b) followed by insertion of a 14 × 55 mm self expandable Schneider Wall Stent. Post stent deployment balloon dilatation using a Medi tech XXL balloon (Boston Scientific, 16 mm × 40 mm) was carried out. (c) Frontal view and (d) lateral view: Final venography demonstrated free flow of contrast across the stent with disappearance of paravertebral collaterals.

Following membranotomy, the catheter was advanced into the right atrium. The wire was exchanged for floppy tipped wire, which was navigated into the subclavian vein. A predilatation of the membranous segment was followed by stenting (Figure 3). Check venogram demonstrated restoration of antegrade venous flow.

## DISCUSSION

Membranous obstruction of the IVC is a significant yet underreported cause of hepatic venous outflow obstruction in Asia.^1,3^ Clinical manifestations vary from asymptomatic cases to advanced hepatic dysfunction, depending on the chronicity and extent of obstruction. Endovascular treatment with balloon angioplasty and stenting, has become the preferred treatment, offering durable results compared to surgical options.^4^

Catheter venacavography is gold standard diagnostic test.^2^ It generally shows the dilated IVC, collateral veins, and a conical obstruction. This configuration forms a silhouette resembling a dolphin in motion on cine fluoroscopy an appearance we term the “Acrobatic Dolphin mimic.” Although not previously described in the context of IVC imaging, this analogy may serve as a useful visual mnemonic for recognizing membranous obstruction.

Cho and Baker (1997) described “The leaping dolphin sign’, in pneumoperitoneum on a supine abdominal radiograph.^5^ Other imaging metaphors the “rat tail” in achalasia, “cobra head” in ureterocele, or “coffee bean” in sigmoid volvulus exemplifies how natural analogies aids diagnosis and mnemonic retention.

Beyond its resemblance, the dolphin mimic highlights a conical, beak-shaped membrane that provides a valuable landmark for endovascular intervention. Schaffner et al. (1967) proposed that the convex contour of the obstruction may result from chronic hemodynamic forces causing the membrane to bow in the direction of blood flow, creating a tapered configuration distinct from abrupt thrombotic occlusions.^6^ In this context, we hypothesize that the central or juxta-central portion of this taper corresponding to the “beak” is structurally weaker than its margins, making it a favorable site for attempting membranotomy. While based on our procedural experience, this observation remains histopathologically unproven.

Wang et al emphasized that puncture through atrial aspect of the taper reduces the risk of entering collateral vessels inadvertently.^4^ However, in our experience, puncturing from the concave hepatic side is advantageous. The narrower lumen within the “beak” offers a stable parking zone for the catheter-wire combination, facilitating controlled puncture of the membrane while aligning with the IVC’s natural course and minimizing collateral injury .

## CONCLUSION

The “acrobatic dolphin mimic” represents a novel and memorable radiological sign in obliterative hepatocavopathy. The conical, beak-shaped taper not only provides a visual clue to diagnosis but also serves as a procedural landmark for safe and effective membranotomy. Incorporating natural analogies into imaging interpretation strengthens both clinical practice and teaching, particularly in regions where hepatocavopathy remains prevalent.
